# Lipid-dependent regulation of the unfolded protein response

**DOI:** 10.1016/j.ceb.2014.12.002

**Published:** 2015-04

**Authors:** Romain Volmer, David Ron

**Affiliations:** 1 Cambridge Institute for Medical Research, University of Cambridge, Cambridge, United Kingdom; 2 Wellcome Trust MRC Institute of Metabolic Science, Cambridge, United Kingdom; 3 NIHR Cambridge Biomedical Research Centre, Cambridge, United Kingdom; 4 Université de Toulouse, INP, ENVT, INRA, UMR 1225, IHAP, Toulouse, France

## Abstract

Protein folding homeostasis in the lumen of the endoplasmic reticulum is defended by signal transduction pathways that are activated by an imbalance between unfolded proteins and chaperones (so called ER stress). Collectively referred to as the unfolded protein response (UPR) this homeostatic response is initiated by three known ER stress transducers: IRE1, PERK and ATF6. These ER-localised transmembrane (TM) proteins posses lumenal stress sensing domains and cytosolic effector domains that collectively activate a gene expression programme regulating the production of proteins involved in the processing and maturation of secreted proteins that enter the ER. However, beyond limiting unfolded protein stress in the ER the UPR has important connections to lipid metabolism that are the subject of this review.

**Current Opinion in Cell Biology** 2015 **33**:67–73

This review comes from a themed issue on **Cell regulation**

Edited by **Jodi Nunnari** and **Johan Auwerx**

For a complete overview see the Issue and the Editorial

Available online 25th December 2014

**http://dx.doi.org/10.1016/j.ceb.2014.12.002**

0955-0674/© 2014 The Authors. Published by Elsevier Ltd. This is an open access article under the CC BY license (http://creativecommons.org/licenses/by/3.0/).

## Lipid regulation of the endoplasmic reticulum unfolded protein response is conserved in eukaryotes

Clues to a lipid connection were provided by the very earliest studies in which UPR components were first identified. The genes encoding what we now know to be the UPR transducer IRE1 (also known as ERN1, for ER to nucleus transducer 1) and its downstream transcription factor HAC1/IRE2 (the yeast ortholog of the metazoan XBP1) were first identified as required for growth in medium deprived of inositol [1,2], an essential building block of yeast phospholipids. Depletion of inositol from growth medium strongly activates IRE1 signalling [3], whereas IRE1 and HAC1 are required for full expression of genes involved in lipid metabolism in yeast [4]. Furthermore, deletion of genes regulating lipid metabolism strongly activates UPR signalling in yeast [5^••^].

Lipid-dependent activation of IRE1 was also observed following loading of yeast with saturated fatty acids and sterol [6^•^] and following deletion of the regulators of sphingolipid synthesis ORM1 and ORM2 [7]. These findings established firm links between lipids and UPR signalling in yeast: the UPR is activated by altered lipid metabolism whose consequences are mitigated by UPR signalling.

In mammalian cells, enhanced UPR signalling has been observed in cholesterol-loaded macrophages [8], in pancreatic beta cells exposed to saturated fatty acids [9] and in cells in which increased lipid saturation was achieved by genetic or pharmacological inhibition of the **Δ**9 desaturase, stearoyl-CoA desaturase 1 [10,11]. Perturbation of sphingolipid metabolism causing increased levels of ceramides also activated the UPR in mammalian cells [12,13]. Increased UPR markers have also been observed in the liver and adipose tissue of mice fed a high fat diet and in severely obese humans [14,15]. These observations indicate that the ability of UPR transducers to sense perturbations to lipid homeostasis is conserved in eukaryotes.

## Linking lipid perturbation to activation of UPR transducers

Lipid composition could modulate protein folding in, or trafficking through, the ER, indirectly activating UPR transducers by changing the level of unfolded proteins. Changes in the lipid composition could, for example, perturb ER calcium homeostasis, inhibiting the function of calcium-dependent enzymes and chaperones. In support of this idea, ER stress signalling in the liver of obese mice correlated with perturbations of ER calcium homeostasis through an inhibition of the SERCA transporter caused by an increase in the ratio between phosphatidylcholine and phosphatidylethanolamine in membranes of the hepatocytes [14]. UPR in cholesterol-loaded macrophages was also linked to inhibition of the SERCA pump [16].

However, there are clues that lipid changes may affect UPR signalling independently of their effect on protein folding in the ER lumen. In yeast, depletion of the phospholipid building block inositol strongly activated IRE1 but had no effect on the mobility of the ER chaperone BiP/KAR2 (BiP mobility is strongly retarded by unfolded protein stress) [17]. This indicates that inositol depletion activates the UPR without causing lumenal unfolded protein stress. In *C. elegans*, deletion of mdt-15, a subunit of the transcriptional regulator complex Mediator, was associated with an increase in membrane lipid saturation and the activation IRE1 and PERK without evidence for concomitant formation of protein aggregates in the ER, suggesting that activation of the UPR stress transducers may have a component that is independent of unfolded protein stress [18^•^].

**Figure 1:**
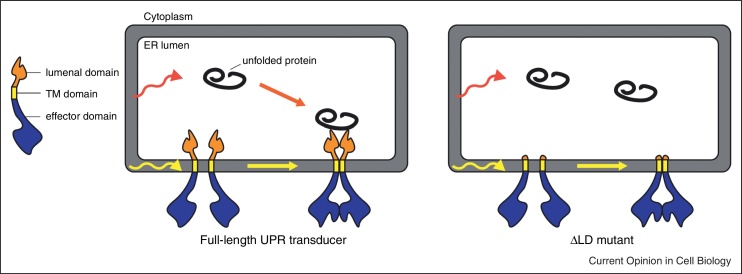
Mechanisms of lipid sensing by the UPR transducers. Perturbations of the ER lipid bilayer composition could impair the folding of ER proteins, thereby activating the full-length UPR transducers via their lumenal unfolded proteins sensing domain (left panel). However, mutant IRE1 and PERK lacking their lumenal domain (**Δ**LD mutants, right panel) are also activated by lipid perturbations in a process that requires ER membrane tethering via a TM domain [19^••^,20^••^]. Lipid-dependent activation of the **Δ**LD UPR transducers is independent of unfolded protein accumulation and likely proceeds by association of the TM domains in response to changes in the biophysical properties of the membrane. Dimerization of the effector domains of IRE1 and PERK (required for their allosteric activation) follows association of the TM domains. In the full-length proteins, stability of the activating effector-domain dimer would be further increased by the dimerization of the lumenal domain of IRE1 or PERK. Response of TM domains to changes in lipid composition of the ER membrane likely modulates a cooperative process involving the dimerization of both the effector and the lumenal domains.

Direct evidence that lipids may activate the UPR independently of their effects on unfolded protein burden in the lumen was provided by the observation that IRE1 and PERK lacking their lumenal unfolded protein stress-sensing domains were activated in yeast deprived of inositol [19^••^] or mammalian cells exposed to saturated fatty acid [20^••^]. Activation of the mutant IRE1 and PERK lacking their lumenal domain required ER membrane tethering via a TM domain [20^••^]. Furthermore, sensitivity to the lipid composition of the membrane bilayer was observed in a reconstituted system composed only of liposomes and a truncated PERK lacking its lumenal domain, but retaining the TM and cytosolic effector domains [20^••^]. Thus, perturbations of ER membrane lipids can directly activate IRE1 and PERK independently of unfolded proteins in a process that requires TM domain insertion into the ER lipid bilayer (Figure 1).

Activation of the UPR signal transducers hinges on the transition from an inactive monomeric state to an active dimer/higher order oligomeric structure (reviewed in [21]). The recent findings obtained with mutant UPR signal transducers lacking their lumenal domains suggest that the TM domain of PERK and IRE1 could promote dimerization by responding to changes in the biophysical properties of the ER membrane [20^••^]. In comparison to other organelles, the ER lipid bilayer is a thin and fluid membrane, characterized by low cholesterol content (for a review of the key differences in the lipid composition of the different organelles, see [22]). Changes in the lipid composition, such as increased acyl chain saturation, are therefore likely to modify the ER membrane biophysical properties and influence the behaviour of TM peptides within the lipid bilayer.

**Figure 2:**
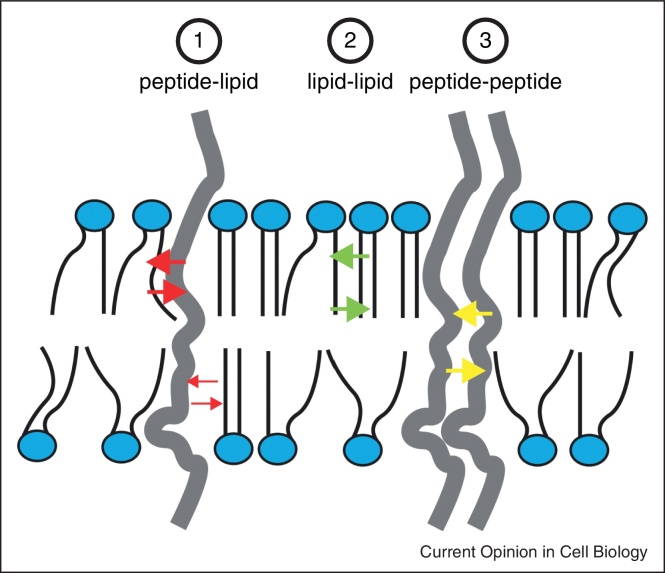
Molecular interactions in the plane of the lipid bilayer. Within the plane of the membrane, a TM helix can interact solely with neighbouring lipids and therefore remain as a monomer, or it can also interact with another TM helix and therefore form a dimer or higher order structure. The monomer/dimer equilibrium is modulated by the strength of the three types of interactions taking place in this simplified model of TM helices in a lipid bilayer: (1) peptide–lipid interaction, (2) lipid–lipid interaction and (3) peptide–peptide interaction. Changes to the lipid composition can affect the strength of these competing interactions and therefore modify the monomer/dimer equilibrium of the embedded TM [56].

In the lipid bilayer, proteins and lipids are subject to three types of interactions: peptide–peptide, peptide–lipid and lipid–lipid (Figure 2). The relative strength of which influences the oligomeric state of a TM peptide in the lipid membrane [23]. The biophysical properties of membranes significantly influence the partitioning of TM peptides between their monomeric and dimeric/oligomeric state. This is played out through several defined mechanisms that might contribute to lipid regulation of UPR signalling, independently of unfolded proteins (Box 1).

A measure of lipid-mediated activation of the mutant IRE1 lacking its lumenal domain was retained when the TM domain of IRE1 was swapped to that of calnexin, an unrelated ER protein, or when the TM peptide sequence was scrambled [20^••^]. These findings suggest that lipid-dependent activation of the UPR transducers has relaxed specificity with respect to protein–protein or protein–lipid interactions involving the TM amino acid side chains, but rather proceeds through generic biophysical mechanisms of dimerization and approximation that are shared by diverse TM domains, described in Box 1. As long as it allows insertion in the ER membrane, the TM domain of the UPR transducers can tolerate a range of amino acid substitutions in its sequence. However, the sensitivity of the assays used to measure the effects of TM swaps on intensity of UPR signalling is rather limited. It thus remains possible that more sensitive assays might reveal sequence constrains on TM domains of the UPR transducers driven by the need to respond to lipids.

It is noteworthy that the response of full-length IRE1 and PERK to lipid perturbation was considerably stronger than their lumenal domain-deleted derivatives [20^••^]. This observation is consistent with an important contribution of unfolded protein stress to lipid-mediated activation of IRE1 and PERK. Alternatively, as the lumenal domain stabilises the dimer [24,25], it may contribute to lipid-mediated activation of the UPR even in circumstances that are not associated with any further increase in unfolded protein stress. Cooperativity in dimerization suggests that perturbations in the ER lipid bilayer composition may lower the threshold for unfolded protein stress-mediated activation and that direct lipid-dependent regulation and conventional lumenal unfolded protein stress mediated activation of the UPR transducers are likely to modulate each other.

## UPR modulation of lipid metabolism

IRE1 and PERK also modulate lipid metabolism, placing the UPR transducers as both sensors of primary lipid perturbations and regulators of lipid homeostasis. In yeast, the importance of IRE1 to lipid metabolism is stressed by the dependence of *IRE1* mutant yeast on exogenous inositol for their survival [1,3]. In the absence of exogenous inositol, yeast IRE1 is required for the expression of *INO1* encoding inositol-3-phosphate synthase, an enzyme catalysing a rate-limiting step in the synthesis of phosphatidylinositol [3,26]. Yeast genes controlling the expression of key enzymes in lipid metabolism are upregulated following induction of the UPR [4]. The role of *IRE1* in regulating phospholipid synthesis is conserved in mammals, where activated splicing of its downstream effector XBP1 has been shown to contribute to ER membrane expansion through the stimulation of the expression of genes involved in phospholipid synthesis [27^•^]. In addition, IRE1 and PERK signalling have been shown to regulate lipid metabolism in vivo [28,29].

Remarkably, lipid perturbations in yeast triggered predominantly compensatory changes affecting protein quality control, contrasting with minimal adjustments to lipid metabolism [30^••^]. Restoration of protein quality control was dependent on IRE1, while expression of lipid metabolism genes previously identified as IRE1 targets remained largely unchanged. In this study, lipid disequilibrium was triggered by genetic deletion of CHO2 or OPI3, two enzymes catalysing respectively the initial and late steps of phosphatidylcholine synthesis from phosphatidylethanolamine. It should be noted that *OPI3* expression is upregulated following IRE1 activation [4,31], raising the possibility that IRE1-dependent compensatory changes in lipid composition might have been blocked by the mutation in *OPI3*.

Following perturbation of the ER membrane lipid composition, compensatory changes to lipid homeostasis and protein homeostasis could be equally important to alleviate cellular stress. Indeed, as discussed earlier, lipid disequilibrium within the ER membrane is likely to affect protein folding within the ER lumen, for example by perturbing ER calcium homeostasis [14]. Lipid perturbations could also cause proteotoxicity by affecting protein folding within the membrane, protein translocation or trafficking, or by causing membrane protein aggregation. Activation of IRE1α and PERK by changes in ER membrane lipid composition may be a mechanism allowing the cell to adapt the flow of protein entering the ER, as well as the folding apparatus in response to changes in the lipid composition that might otherwise promote unfolded protein accumulation.

## Physiological significance of lipid activation of the UPR

Activation of the UPR and perturbations in the ER lipid composition are observed in morbid obesity [15,32]. Moreover, UPR activation has been linked to the development of insulin resistance or beta-cell death in morbid obesity [9,14,33]. Altogether these observations suggest that lipid-dependent activation of the UPR transducers could contribute to the pathogenesis of morbid obesity.

Flaviviral non-structural proteins are ER membrane associated proteins triggering membrane rearrangements [34]. Though they lack substantial ER lumenal domains, they have been shown to activate the UPR, suggesting that lipid-dependent activation of the UPR could operate in Flavivirus infected cells [35–37]. Lipid-dependent activation of the UPR transducers could also occur during cellular processes marked by a discrepancy between the level of UPR activation measured and the level of unfolded proteins detected in the ER, such as B lymphocyte development [38–40]. In line with this hypothesis, modifications of the ER lipid membrane could also initiate what has been called anticipatory ER stress [41,42] in which the UPR is triggered independently of unfolded proteins, thereby allowing the cells to adapt their ER folding capacity in anticipation of unfolded protein stress.

Gaging the physiological or pathological significance of lipid-mediated activation of the UPR transducers represents a major challenge for the future. Currently, direct lipid-dependent activation is isolated from any affects of unfolded protein stress by the expression of mutant UPR transducers lacking their lumenal domain. Unfortunately this technique cannot be readily applied to study physiological circumstances, such as those listed above.

## Conclusions and perspective

The proposed tuning of UPR signalling by lipids, mediated by simple biophysical principles, could represent an addition strand in the lipid-UPR dialectic; the physiological significance of which remains to be explored. Major lipid perturbations are found alongside UPR activation in important pathophysiological circumstances such as viral infection and severe obesity, and these would be good candidates for testing the biological role of the lipid-UPR dialectic.

The highly cooperative nature of IRE1 (and likely PERK) activation is poised to respond to subtle variation in the factors that alter the tendency of TM proteins to dimerize. Acting alone, each of these simple biophysical principles would probably have only weak effects on protein dimerization. This may account for the considerable redundancy in the sequence requirements for a functional IRE1 TM domain [20^••^]. However, we propose that in association with dimerization-competent lumenal and cytosolic domains that respond to other cues, these weak forces acting on the TM domains might allow lipids to tune UPR signalling.

Changes in membrane properties are likely to influence other TM proteins by such generic mechanisms. The identification of such proteins and a better understanding of the biophysical parameters of the membrane that govern the modulation of their function represent an interesting challenge for cell biology.

## References and recommended reading

Papers of particular interest, published within the period of review, have been highlighted as:

• of special interest
•• of outstanding interest

## Box 1 Biophysical principles regulating TM–TM dimerization

**Hydrophobic mismatch**

Stable membrane integration of single-pass proteins is favoured by matching TM length to the thickness of the lipid bilayer, as evidenced by the gradual increase in TM length of single pass resident proteins that matches the increase in the lipid bilayer thickness along the secretory pathway [43^••^]. However, a mismatch can occur when changes to lipid composition causes the thickness of the lipid bilayer to be different from the length of the hydrophobic TM peptide. The thickness of the lipid bilayer depends on the length of the acyl chains and on the degree of their unsaturation. The less flexible saturated acyl chains remain in an extended conformation and therefore tend to increase the thickness of the lipid bilayer [44].

As lipid bilayer thickness increases, polar amino acid side chains that would otherwise be located at the water–lipid interface are forced into the hydrophobic lipid bilayer. Their presence in the lipid bilayer creates a thermodynamically unfavourable situation, which can be relieved by tilting or by the formation of hydrogen bonds or salt bridges between polar side chains from adjacent TMs, favouring dimerization [45–47].

**Acyl chain flexibility**

The degree of phospholipid unsaturation can also influence TM–TM interactions, independently of effects on the thickness of the lipid bilayer. Saturated acyl chains are less flexible than unsaturated acyl chains. As a consequence saturated acyl chains interact less efficiently with the surface of the TM helices than unsaturated acyl chains. Weakening of TM–lipid interaction by acyl chain saturation favours the competing TM–TM interaction thereby promoting dimerization [48].

**Lipid microdomains**

Biological membranes composed of heterogeneous lipids can segregate into lipid microdomains, such as liquid ordered domains and liquid disordered domains [22,49]. TM proteins preferentially localize into different membrane domains where their local density increases [50,51]. Changes in lipid composition can modify the relative size of these membrane domains. As a consequence, the local concentration of a TM protein that partitions asymmetrically between lipid domains, may change, thereby affecting the monomer–dimer equilibrium [48,52]. In comparison to the plasma membrane, the relative low cholesterol and sphingolipid content of the ER makes the formation of liquid ordered microdomains unlikely. However, this observation does not exclude the possibility that microdomains with specific lipid composition are formed in the ER, either locally at the site of sphingolipid or cholesterol synthesis [53], or in the spatially distinct sub-compartments of the ER (sheets, tubules, ER exit sites, mitochondrial associated membrane) [54,55].
